# A methyl-sensitive element induces bidirectional transcription in TATA-less CpG island-associated promoters

**DOI:** 10.1371/journal.pone.0205608

**Published:** 2018-10-17

**Authors:** Amin Mahpour, Benjamin S. Scruggs, Dominic Smiraglia, Toru Ouchi, Irwin H. Gelman

**Affiliations:** 1 Department of Cancer Genetics, Roswell Park Comprehensive Cancer Center, Buffalo, NY, 14263, United States of America; 2 Epigenetics and Stem Cell Biology Laboratory, National Institute of Environmental Health Sciences, Research Triangle Park, NC, 27709, United States of America; Bar-Ilan University, ISRAEL

## Abstract

How TATA-less promoters such as those within CpG islands (CGI) control gene expression is still a subject of active research. Here, we have identified the “CGCG element”, a ten-base pair motif with a consensus sequence of TCTCGCGAGA present in a group of promoter-associated CGI-enriched in ribosomal protein and housekeeping genes. This element is evolutionarily conserved in vertebrates, found in DNase-accessible regions and employs RNA Pol II to activate gene expression. Through analysis of capped-nascent transcripts and supporting evidence from reporter assays, we demonstrate that this element activates bidirectional transcription through divergent start sites. Methylation of this element abrogates the associated promoter activity. When coincident with a TATA-box, directional transcription remains CGCG-dependent. Because the CGCG element is sufficient to drive transcription, we propose that its unmethylated form functions as a heretofore undescribed promoter element of a group of TATA-less CGI-associated promoters.

## Introduction

Gene expression is one of the most critical, yet enigmatic, biological processes that defines cellular and organismal identity, and that mediates cellular response to internal and external stimuli [1]. Importantly, dysregulation of this process is known to contribute to various human diseases such as cancer [2]. With the discovery of RNA polymerases, the mechanisms of how transcription occurs have been extensively studied in many organisms [3]. In contrast to the relatively simple prokaryotic transcriptional system, metazoan transcription is considerably more elaborate and involves complicated promoter structures, multiple functional DNA elements and a repertoire of specific general transcription factors. These factors and DNA elements are required to facilitate accurate transcriptional initiation, elongation, and termination [4–6].

The best-known DNA element that mediates the initiation of transcription of protein-coding genes is the TATA box with the consensus sequence TATAA [7]. This element is usually located 25 to 34 base pairs upstream of transcription start sites (TSS). However, many human promoters, including those regulating housekeeping genes lack this DNA element [8], suggesting that TATA-less promoters are controlled by different yet poorly understood mechanisms. A few novel elements have been described that presumably function as core promoter elements in TATA-less promoters [9–12]. Yet, most of these promoter elements (e.g. GC-box or Inr motif) require additional transcriptional activator binding sites in order to drive efficient directional transcription.

Vertebrate genomes contain short GC-rich sequences that are typically less than 1 kb long, termed CpG islands (CGIs) [13, 14]. These regions are considered to be critical for transcriptional regulation of a large group of genes that include housekeeping genes [15]. Most CGI-associated promoters lack a TATA-box yet contain “GC-box” binding sites for the general transcription factor SP1 although these GC boxes are not sufficient to induce transcription on their own [15–18]. CGI-associated promoters typically induce bidirectional transcription that produces coding and non-coding transcripts [19, 20]. Thus, depending on the stability of the non-coding RNA, CGI-associated promoters can generate more stable long non-coding RNAs (lncRNA) or short-lived transcripts [21]. To date, no specific independently-acting promoter element governing these CGI-associated bidirectional promoters has been described.

In this study, we analyzed DNase-accessible CGIs in the K562 cell line and found an enriched motif with the consensus sequence of TCTCGCGAGA, which we termed the “CGCG element” due to the characteristic invariable core sequence. This element confers transcriptional activity independent of other transcriptional activator sequences. Promoter sequences related to the CGCG element have been reported previously for several individual genes, but their functional significance was never explored [22–25]. A genome-wide computational study identified a similar motif among those most enriched in human promoters, suggesting a possible functional role [26]. Our data indicate that the CGCG element is enriched in TATA-less CGI-associated promoters and evolutionarily conserved among vertebrates. Importantly, it is associated with bidirectional transcription only in the context of CGI-associated promoters as assessed by analysis of GRO-Cap and Start-seq datasets that identify sense versus anti-sense TSS-associated nascent transcripts. Using novel reporter constructs, we demonstrate that the CGCG element suffices as a promoter element to drive bidirectional transcription. Gene Ontology analysis indicates that this element is enriched in the promoters of housekeeping genes, most notably those controlling RNA metabolism and translation, and of long non-coding RNAs. Together, our results indicate that the CGCG element functions as a previously unknown driver of CGI-associated TATA-less promoters.

## Materials and methods

### Cell culture and treatments

Human embryonic kidney 293T and mouse NMuMG cells were cultured in Dulbecco’s Modified Eagle Medium (DMEM) media supplemented with 10% fetal bovine serum, penicillin and streptomycin at 37°C and 5% CO2. For the *α*-amanitin treatment experiment, HEK293T cells were transfected with SV40 promoter-driven firefly luciferase reporter (pGL2-pro), or a construct containing a copy of TCTCGCGAGA. 24h post-transfection, cells were treated with 5 *μ*g/ml *α*-amanitin (Santa Cruz Biotechnologies) as described [27] or with PBS (control), and firefly and Renilla luciferase bioluminescence activities were measured 24h after treatment.

### Reporter constructions and assays

One to three copies of the CGCG elements from the *DENR* promoter were synthesized as double stranded oligonucleotides (IDT DNA) and cloned into the BglII and MluI restriction sites of a luciferase reporter construct that lacks promoter sequences (pGL2-basic, Promega). 1 *μ*g of cloned reporter DNA along with 100 ng of a Renilla luciferase reporter construct (pRL-TK) as transfection control were transfected into HEK293T using X-tremeGENE 9 (Roche) reagent according to manufacturer’s protocol. The luciferase activities were measured 24h after transfection according to the Dual Luciferase assay protocol (Promega).

The bidirectional luciferase (Empty-LuBiDi) reporter was constructed by PCR amplification and subsequent cloning of the firefly luciferase gene from pGL2-Basic into the BglII site of the promoterless Renilla luciferase cassette from the pRL-Null plasmid followed by site-directed mutagenesis to remove secondary the BglII recognition site downstream of firefly luciferase poly-A site. The primer sequences used are described in S1 File. Bioluminescence assays were performed as described above except that transfection was normalized using a co-transfection vector that expresses secretory alkaline phosphatase (pSELECT-zeo-SEAP, Invivogene).

For the construction of the bidirectional fluorescence reporter, pmCGFP, we first PCR amplified and cloned the h2b-mCherry fused gene (plasmid Addgene id #20972) head-to-head into a promoterless eGFP containing construct. The resulting construct (eGFP + h2b-mCherry) was then digested with AgeI to release h2b-coding fragment and self-ligated to generate the pmCGFP (eGFP + mCherry). Double stranded oligonucleotides encoding one or three copies of TCTCGCGAGA were spliced into the AgeI restriction site of this reporter.

For CpG-free reporter and methylation experiments, an oligonucleotide encoding a single copy of TCTCGCGAGA was inserted into the HindIII restriction site of pCpGfree-basic-Lucia (Invivogen). 10 *μ*g of purified plasmid was incubated at 37°C for 8h with 10 enzymatic units (U) of M.SssI methyltransferase (NEB) supplemented with fresh 100 *μ*M S-adenosyl methionine (SAM) as the methyl donor. DNA was extracted using phenol-chloroform followed by ethanol precipitation. The DNA was subjected to another an additional 8h incubation with M.SssI was followed by DNA extraction. As a control, a mock reaction was also carried out lacking M.SssI enzyme. To test the methylation efficiency, 300ng of DNA was digested with 10 U of NheI and BstUI for 30 min at 37°C. Because CGCG methylation blocks BstUI cleavage, empty vector or and methylated constructs digested only by NheI enzyme producing two indistinguishable bands at 2.4 kb. However, unmethylated TCTCGCGAGA which is cut by BstUI enzyme as well as NheI produced three smaller bands.

The sequences of inserts for each promoter fragment and related mutations are provided in the S1 File.

### qRT-PCR

HEK 293T cells were transfected with 1 *μ*g of LuBiDi reporter DNAs containing 0, 1, 2, 4 copies of TCTCGCGATA. Cells were lysed after 24h using TRIzol (Life Technologies), RNA was extracted using chloroform-isopropanol, and after resuspension, incubated with RNase-free DNase I (Thermo Fischer Scientific) followed by extraction by acidic-phenol chloroform protocol, precipitation using ethanol and resuspension in RNase-free water. 1 *μ*g of the resulting purified RNA was used to prepare cDNA using M-MLV reverse transcriptase according to manufacturer’s recommended protocol (Life Technologies). Transcript levels were measured using iTaq Universal SYBR Green Supermix (Bio-Rad) on an ABI-7900 RT-PCR instrument. Transcript levels were normalized using primers for *HPRT1*. Primers designed to amplify the bacterially-expressed ampicillin resistance gene in the LuBiDi construct were used as a negative control to rule out plasmid contamination. Melting curve analyses for all PCR experiments were performed to validate faithful amplification of PCR products. Information on primer sequences is described in S1 File.

### Chromatin immunoprecipitation (ChIP)

ChIP was performed according to a protocol described in Lee et al. [28]. In brief, 10 million HEK293T cells were cultured in 15 cm dishes and transfected with 10 *μ*g reporter DNA using X-tremeGENE 9 transfection reagent. 48h post-transfection, cells were treated with the cross-linking reagent formaldehyde (1% in PBS, Sigma) for 5min. Cross-linking was stopped by adding glycine solution (0.125 M) for 10min, followed by 2 washes with ice-cold PBS. The cells were lysed in buffer A (50 mM HEPES-KOH, pH 7.5, 140 mM NaCl, 1 mM EDTA, 10% glycerol, 0.5% NP-40, 0.25% Triton X-100, 1X protease inhibitors [Roche]), and their nuclei were isolated by centrifugation (5 min, 1000 RPM) and lysed by sonication in buffer B (10 mM Tris-HCl, pH 8.0, 100 mM NaCl, 1 mM EDTA, 0.5 mM EGTA, 0.1% Na-deoxycholate, 0.5% N-lauroylsarcosine, 1X protease inhibitors) using a Biorupter(Diagenode), specifically, five rounds of 10sec sonication to obtain 300-600bp range chromatin fragments. The resulting sheared chromatin was immunoprecipitated (IP) using 20 *μ*g of antibody against Pol II (Santa Cruz, N-10) or a non-specific Isotype Mouse IgG as a mock control (Santa Cruz). The IP complexes were then bound to Protein A/G Dynabeads and washed five times using RIPA washing buffer (50 mM HEPES-KOH, pKa 7.55, 500 mM LiCl, 1 mM EDTA, 1.0% NP-40, 0.7% Na-deoxycholate). The DNA was extracted from the beads using buffer C (50 mM Tris-HCl, pH 8.0, 10 mM EDTA, 1.0% SDS) and quantified by qPCR using primers designed to amplify the promoter region of the reporter construct.

### Fluorescent microscopy and live imaging

1 *μ*g of pmCGFP reporter constructs containing 0, 1, 2 copies of TCTCGCGAGA along with a CMV promoter-driven Blue Fluorescent Protein expression plasmid (CMV-BFP) were transfected into HEK293T cells. Images were taken 24h post-transfection using a Nikon Eclipse TE2000-E fluorescence microscope. For live imaging, images were taken every 30min with an exposure time of 1sec immediately after reporter transfection for 24h in an incubating chamber supplied with humidity and 5% CO2. 16-bit Tiff images from individual channels were used to generate MOV files using Videomach software (http://gromada.com/videomach/). The final video was produced using Adobe Premiere CC 2017.

### Double nickase Cas9-mediated genome editing of *DENR* promoter

Short guide RNAs (sgRNAs) to target *DENR* promoter were designed using the MIT CRISPR sgRNA design tool (http://crispr.mit.edu/). Double-stranded oligonucleotides representing sgRNAs (S1 File) were then cloned into pSpCas9 (BB)-2A-GFP (PX458) and pSpCas9 (BB)-2A-Puro (PX459) V2.0 (Addgene plasmids 48138 and 62988). Constructs were then co-transfected into HEK293T cells and 24h later selected for puromycin resistance (3 *μ*g/mL) for another 72h. GFP-expressing single cells were sorted using an Aria II FACS and incubated in 96 well dishes for two weeks to form visible cellular clones. DNA was extracted from the clones using QuickExtract solution (Epibio), and successful deletions were confirmed by Sanger sequencing of PCR products. Ribbon sequences were produced using the pyRibbon software which we deposited in https://github.com/AminMahpour/pyRibbon/.

### Lentivirus mediated knockdown of ZBED1 and immunoblotting

For ZBED1 knockdown, lentiviruses targeting ZBED1 or non-targeting (pLKO.1) were purchased from Sigma (MISSION shRNA). Cells were transduced with the virus according to the manufacturer’s protocol (Sigma) and transduced cells were selected for puromycin at 3*μ*g/mL for four days.

For immunoblots, cells were lysed in NET-N buffer (100 mM NaCl, 20 mM Tris-HCl pH 8.0, 0.5 mM EDTA, 0.5% NP-40) supplemented with protease inhibitors cocktail at 4°C. In all experiments, 20 *μ*g of total proteins/lane were analyzed by SDS-PAGE followed by blotting as described Previs et al. [29]. Antibodies included those specific for DENR (Santa Cruz #22), ZBED1 (Santa Cruz H-9) GFP (Santa Cruz B-2), mCherry (Abcam 1C51) or alpha-tubulin (Santa Cruz A-6) as a loading control.

### Oligonucleotide pull-down assay

Kaiso binding to CGCG elements was assessed using biotin-tagged DNA duplexes of unmodified TCTCGCGAGA, TCTCTCGAGA or completely methylated (TCTmeCGmeCGAGA). 10 *μ*M aliquots of each duplex was bound to 100 *μ*L streptavidin Dynabeads as recommended by the manufacturer (Invitrogen). HEK293T cells were lysed using NET-N buffer containing protease inhibitors cocktail (Sigma) and incubated on ice for 30 min. Lysates were centrifuged at 12000 RPM for 10 min to pellet cellular debris, and supernatant representing 500 *μ*g protein was mixed with duplex-charged beads and incubated at 4°C overnight. The beads were washed five times with NET-N buffer, incubated with 50 *μ*L Laemmli loading buffer (1X: 0.02% w/v bromophenol blue, 4% SDS, 20% glycerol, 120 mM Tris-HCl, pH 6.8) and boiled for 5 min to elute bound proteins. The proteins were analyzed by immunoblotting for Kaiso (Santa Cruz #D-10).

### Rapid amplification of cDNA ends (5′-RACE)

To determine divergent TSSs, we transfected near confluent HEK293T cells in 10 cm dishes with 5 *μ*g LuBiDi construct along with 0.5 *μ*g pEGFP-C1 as a transfection control. RNA was extracted 72h after transfection, and the quality and purity evaluated using an Agilent 2100 Bioanalyzer, with samples with RNA integrity number (RIN) values >= 8.0 selected for further analysis. The SMARTer 5′-end RACE (Clontech) protocol was used to determine divergent TSSs from 10 *μ*g of total RNA. Briefly, the RNA was first reverse-transcribed at 42 C for 90 min using poly-dT primers and extended beyond TSS using RT-mediated template switching that employs the SMARTer IIA oligonucleotide only when the 5′-cap is encountered. The resulting cDNA products were amplified using specific internal primers for either firefly or Renilla luciferases plus the Clontech Universal Primer Mix (UPM). A GFP primer set was used as an internal control (S1 File). The PCR products containing TSS were directionally cloned into the linearized pRACE vector using the In-fusion HD system, and individual bacterial clones were obtained following transformation of the ligated products into Stellar competent cells. Sanger sequencing of the resulting plasmid clones (using M13 primer) was used to identify TSSs.

### Motif discovery

The CpG island annotation track in the human genome (hg38) was downloaded from the UCSC genome browser (https://genome.ucsc.edu/), and sequences that overlap with K562 DNase-seq peak track extracted using Bedtools [30]. The resulting sequences were used for motif discovery using the findMotifgenomewide script in the Homer bioinformatics software suite using default command line arguments for the human genome [31].

### Genomic annotation and metagene analysis

The scanMotifgenomewide script from the Homer program version 4.8 was used to locate all instances of motif 7 and 10 in human (hg38) and mouse (mm9) genomes. The annotatePeaks script (Homer) was used to identify motif co-occurrence, genomic annotations, metagene, and enrichment analysis.

### ENCODE conservation, DNase-seq, GRO-Cap, WGBS data analysis

Processed data points for hg38 were extracted and processed using Wigman software for 50 bp upstream and downstream windows for each motif occurrence. For ENCODE WGBS (accession number ENCFF867JRG). The PhyloP and PhastCons conservation scores for hg38 assembly were downloaded from the UCSC genome browser (http://hgdownload.cse.ucsc.edu/downloads.html). ENCODE accession number ENCFF867JRG was used for K562 DNase-seq data. The GRO-Cap dataset for K562 and GM12878 cell lines with GEO accession number of GSM1480321 was used to analyze nascent transcripts in promoters. Pol II ChIP-seq from K562 cell line with the accession number of ENCFF000YWS was used to determine Pol II occupancy state on CGCG elements. Heatmap plots were generated using the in-house written Wigman software (https://github.com/AminMahpour/Wigman).

### CAGE-seq analysis of cells treated with DAC/DMSO

CAGE-seq data derived from DAC/DMSO treated cells was published by brocks et al. [32]. The raw sequencing data (GSE81322) were aligned using the Bowtie2 software on the hg38 reference genome. Average mapped CAGE-seq reads were plotted ± 1kb relative to CGCG elements.

### Start-seq analysis

Start-seq datasets from mouse bone-marrow derived macrophages were published previously and is available for download from the GEO website (GSE62151, https://www.ncbi.nlm.nih.gov/geo/). Data were analyzed as follows: briefly, reads were aligned uniquely to the mm9 genome allowing a maximum of two mismatches with Bowtie version 0.12.8 (-m1 -v2). Sense and divergent TSS were assigned as defined above. Start-seq heat maps depict Start-RNA reads in 10 bp bins at the indicated distances with respect to the TSS. Heatmap plots were generated using Partek Genomics Suite version 6.12.1012.

Individual CGCG element occurrences were identified with FIMO [33]. A ±1 kbp window around TSSs was scanned with a position weight matrix for the CGCG motif with a p-value threshold of 0.001. Motif occurrences were mapped with respect to TSS locations using custom scripts and counted in 10-mer bins. Composite Metagene distributions were generated by summing motifs at each indicated position with respect to the TSS and dividing by the number of TSSs included within each group.

### Gene ontology and gene network analysis

Bedtools Closest feature was used to compile a list of genes with annotated TSS less than 500 bp from CGCG elements on both plus and minus strands from the latest hg38 GTF annotation file (http://www.ensembl.org/info/data/ftp/index.html). A custom script was written and used to determine the number of CGCG elements in annotated coding, non-coding, uni- and bi-directional CGI promoters. Gene Ontology (GO) analysis performed using the GOrilla gene enrichment analysis platform. A list of CpG islands-associated genes was used as the background genes for enrichment analysis [34]. GO enrichment score is defined as (*b*/*n*)/(*B*/*N*) where N is the total number of background CpG island-associated genes that have a GO term, B is the number of genes associated with a specified GO term, n is the number of genes whose promoter contain CGCG element and b is the number of genes in the intersection. Gene set interaction networks were generated and analyzed using REACTOME package v53 (http://www.reactome.org/). Networks were visualized graphically using Cytoscape software version 3.5 (http://www.cytoscape.org/).

### Statistical analysis

All plots were generated and analyzed using GraphPad Prism version 7. Unless noted otherwise, all statistical analyses were performed using Student t-test. The following p-values are presented as *: *p* < 0.05, **: *p* < 0.01, *** : *p* < 0.001, **** : *p* < 0.0001. Error bars represent standard deviation (S.D) from the mean.

## Results

### Motif discovery in DNase-sensitive CpG islands

**Fig 1:**
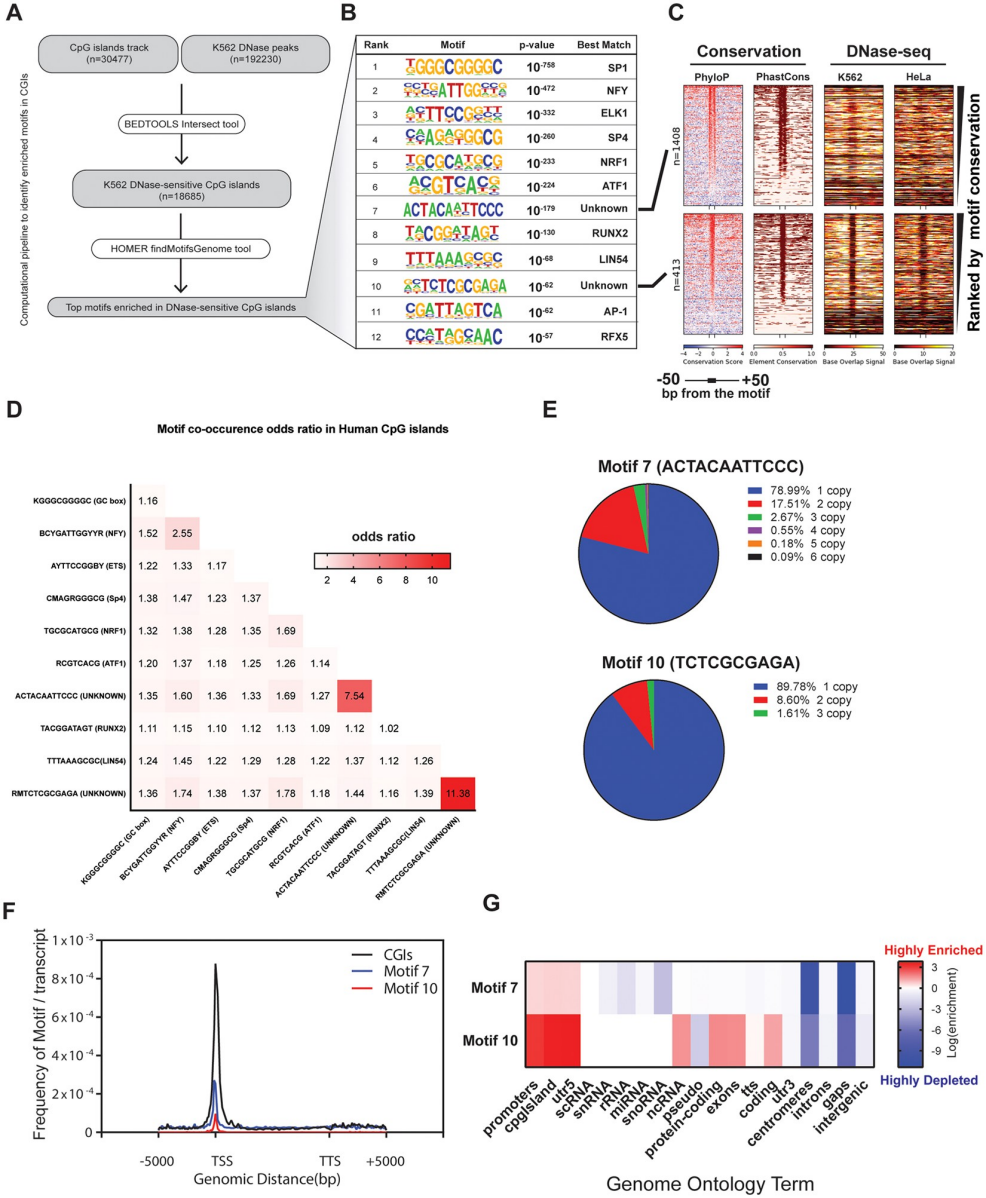
Identification of enriched motifs in human CGIs. A) The computational pipeline used to identify the most enriched motifs in the DNase-accessible CGIs from K562 cells. Bedtools was used to identify CGIs that overlap with ENCODE derived DNase-sensitive peaks. Homer was then used to identify the most enriched DNA sequence motifs in the DNase-accessible CGIs. B) Known transcription factor binding sites in the enriched motif list. C) Heatmaps showing vertebrate conservation and DNase-seq profiles for motifs #7 and #10 including their flanking 50 bp. PhyloP scores represent conservation of individual base-pairs throughout vertebrate genomes. Positive scores in PhyloP heatmaps, shown by red colors, indicate high sequence conservation whereas negative scores (blue colors) indicate acceleration of base pairs. PhastCons heatmaps show probability scores (ranging from 0 to 1) of conserved DNA elements. DNase-seq heatmaps of K562 and HeLa cells indicate DNase accessibility. Low accessibility, as indicated in dark colors, identifies a central DNase-seq footprint associated with motif #10 in both cell lines. Both motifs occur in DNase-sensitive CGIs of many different cell lines, but only motif #10 was consistently associated with a DNase-seq footprint (Fig A in S1 Fig). D) Motif co-occurrence odds-ratio matrix in DNase-sensitive CGIs. The odds-ratio is the value of observed-to-expected coincidence if motifs were distributed by chance. Higher values indicate a higher likelihood of co-occurrence of indicated motifs in the matrix. E) Pie charts indicate frequency of the motif copy number in human CGIs. F) Metagene profiles for all human CGIs, motifs #7 and #10 were generated by the Homer annotated script, showing that both motifs are associated with TSSs. G) Homer annotated script was used to generate genomic annotation enrichment scores to identify genomic regions where these motifs are enriched. The data indicate that motif #7 and #10 are significantly enriched in promoters and CGIs. The enrichment values were calculated using the cumulative hypergeometric distribution method.

Previous analysis suggested that roughly 50 percent of human promoters are associated with a CGI [35]. To identify novel CGI-associated, independently-functioning promoter elements that potentially drive transcription in human CGIs (∼30k), we extracted CGI sequences that overlapped with DNase-accessible regions (∼192k DNase-seq peaks) in the K562 cell line. We then performed an unbiased motif discovery to identify top enriched motifs in transcriptionally active CGI-associated promoters (Fig 1A and 1B). As expected, the SP1 binding site (GC box) had the highest enrichment score consistent with its purported role in driving TATA-less promoters. Binding sites for NRF and ETS were also identified, consistent with roles for these transcription factors in the regulation of CGI-associated housekeeping genes [36]. We also identified two novel sequence motifs (numbered 7 and 10) that were highly conserved within vertebrates (Fig 1C). There were more than 400 incidences of motif #10 that coincided with DNase-seq footprints in multiple cell lines (K562 is shown), suggesting that this motif represents a shared regulatory element (Fig 1C and A in S1 Fig).

Although most CGI-associated promoters contain one copy of the motifs shown in Fig 1E, motifs 7 and 10 tend to occur in multiple copies in a given promoter (Fig 1D and 1E). Genome ontology and metagene profile analyses showed that motifs 7 and 10 are enriched significantly in annotated human CGI-containing promoters, with motif 10 being far more enriched in promoters of annotated coding and non-coding genes despite being less frequent (Fig 1B; motif 7 = 1408 copies vs. motif 10 = 413 copies) (Fig 1F and 1G).

### CGCG elements recruit transcriptional machinery and activate gene expression

**Fig 2:**
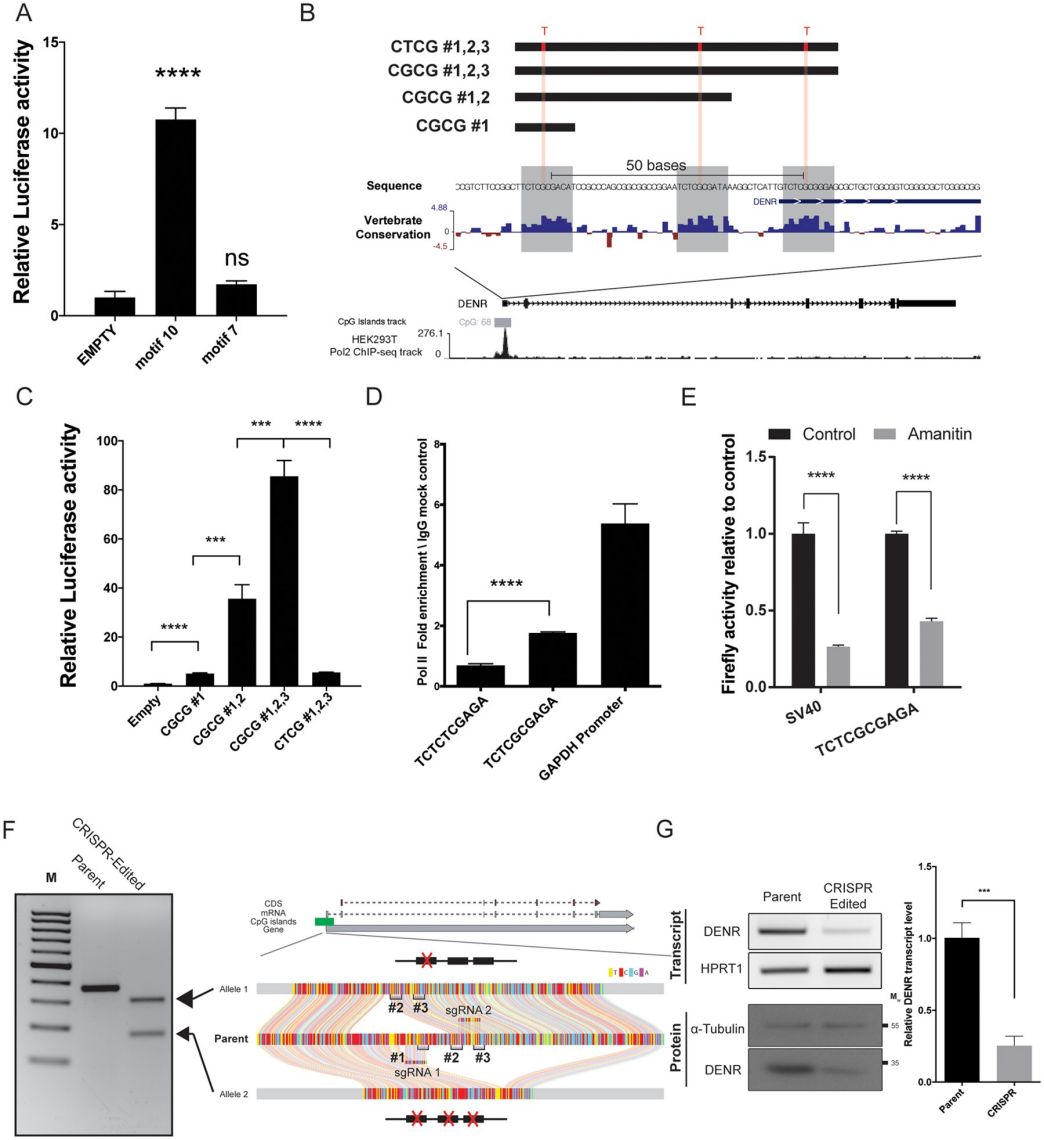
CGCG elements recruit RNA polymerase 2 and activate reporter expression. A) Firefly reporter activity driven by motif 7 and 10. B) The structure and sequence of the human DENR promoter and promoter fragments used for reporter studies. Note that the sequence selected from this promoter do not contain a known core promoter element. The DENR promoter contains three highly conserved copies of the CGCG element. ENCODE Pol II ChIP-seq data from HEK293T cells (bottom) demarcates Pol II occupancy in the promoter region. C) Reporter activity of the corresponding DENR fragments as described in section b. D) Pol II-ChIP on chromatin from HEK293T cells transfected with reporter constructs driven by the wild-type (TCTCGCGAGA) or mutant (TCTCTCGAGA) CGCG motifs, using the human GAPDH promoter as a positive control. E) The effect of *α*-amanitin on TCTCGCGAGA-driven firefly reporter expression. *α*-amanitin significantly reduced both SV40 and CGCG-driven reporter expression. F) CRISPR/Cas9 double-nickase strategy was employed to target CGCG elements in the endogenous DENR promoter. The agarose gel image on the left side of this panel shows genomic PCR amplification products of the DENR promoter in parental and CRISPR-edited cells. Two shorter PCR products associated with CRISPR-edited clone indicate deletions in different alleles. Ribbon plots reveal Sanger sequences of parental and edited alleles shown in the gel image. G) qRT-PCR (“Transcript”) and immunoblot (“Protein”) analyses of DENR expression from the parental and edited cell lines, with the data from three replicate qRT-PCR experiments quantified at right (+/- SD).

To determine whether motif 7 and 10 could confer transcriptional activity independently, we cloned the sequence of the most common variant of each motif (ACTACAATTCCC and TCTCGCGAGA, respectively) into a promoterless construct that encodes a firefly luciferase reporter gene (Empty pGL2-basic). The resulting constructs were then separately cotransfected along with a control reporter for Renilla luciferase driven by the HSV-1 thymidine kinase promoter (pRL-TK) into human embryonic kidney (HEK293T) cells. Motif 10, but not Motif 7, significantly activated firefly reporter gene expression (Fig 2A). This result encouraged us to focus on motif 10, which we named the “CGCG element” based on its central motif. A genome-wide analysis found that this element maps within 50bp of annotated TSSs in human and mouse genomes (Fig B in S1 Fig) suggesting that this element could potentially function to regulate gene expression [37]. To address the function of a specific naturally-occurring CGCG element, we analyzed the CGI-containing promoter of the human Density Regulated gene (*DENR*). The *DENR* promoter contains three tandem CGCG elements separated by 21 and 11 nucleotides (Fig 2B). To determine the role of each CGCG element in this promoter, we inserted promoter fragments containing CGCG #1, CGCG #1,2 and CGCG #1,2,3 into the promoter-less pGL2-basic construct. Although a single copy of the CGCG element significantly increased reporter activity, there was a 7- and 17-fold increase in reporter activity with the addition of the second and third CGCG elements, respectively. Introducing G to T mutations in all CGCG elements (CTCG #1,2,3) dramatically decreased promoter activity, suggesting that the CGCG element is necessary and sufficient to drive reporter expression and that there is a cooperativity between multiple CGCG elements (Fig 2C).

To determine if CGCG element-driven gene expression is dependent on RNA polymerase II (Pol II), we transfected HEK293T cells with reporter constructs that contain either the consensus motif (TCTCGCGAGA) or a G→T transversion mutation (TCTCTCGAGA) and performed chromatin immunoprecipitation (ChIP) for RNA Pol II [38]. As shown in Fig 2D, Pol II bound the wild-type (WT) CGCG but not to the mutant CTCG site. Analysis of the Pol II ChIP-seq ENCODE dataset in HEK293T cells identified binding peaks coincident with the *DENR* promoter containing CGCG elements (Fig 2B). *α*-amanitin, a Pol II inhibitor [39], decreased CGCG element-driven reporter expression (Fig 2E), suggesting that Pol II is indispensable for CGCG dependent gene expression.

To assess the effect of removing CGCG elements on the endogenous *DENR* promoter activity, we employed a CRISPR/Cas9 double-nickase strategy [40] to delete a small CGCG-containing *DENR* region in the HEK293T cell line. One cell clone, containing a deletion of approximately 200 base pairs (bp) removed all three CGCG elements in one allele, and a separate 100bp deletion removed one of the CGCG elements in the other allele without affecting the remaining CGI in the promoter (Fig 2F). Removal of these CGCG-containing regions resulted in a significant decrease in *DENR* transcript and protein levels compared to those in WT controls (Fig 2G). Together with the reporter analyses, these findings suggest that CGCG elements actively recruit transcriptional machinery and promote gene expression in the CGI-associated promoter of *DENR* gene.

### CGCG element confers bidirectional transcription activity in bidirectional reporter constructs

**Fig 3:**
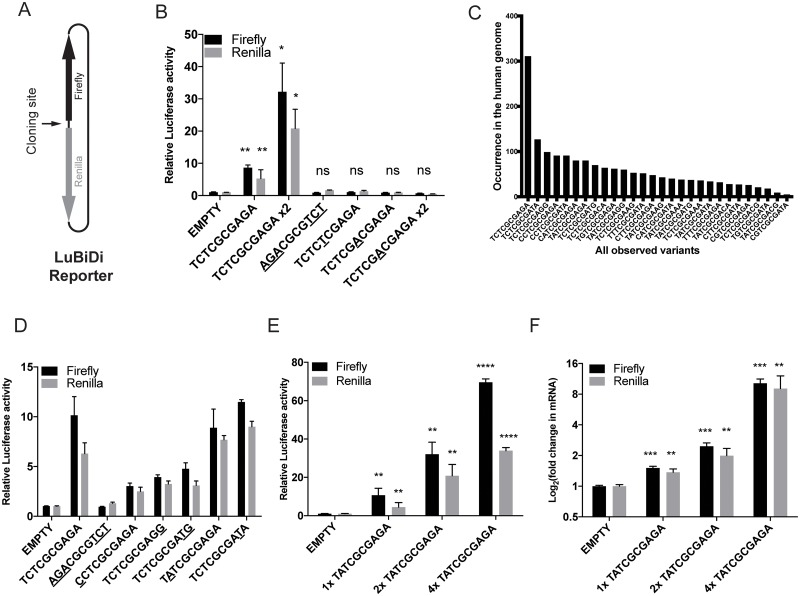
CGCG elements promote bidirectional gene expression in the LuBiDi reporter system. A) The structure of the LuBiDi reporter construct. B) One copy of the TCTCGCGAGA motif inserted in the LuBiDi construct was sufficient to activate the expression of both firefly and Renilla reporters. Flank-exchanged (AGACGCGTCT), G→T transversion mutation (TCTCTCGAGA), or an insertion mutation in the middle of CGCG (TCTCGACGAGA) abolished the dual activation. C) The frequency of common CGCG element sequence variants in the human genome. D) The bidirectional promoter activity of selected naturally-occurring CGCG element variants. E) The effect of CGCG element copy number (TATCGCGAGA motif) in LuBiDi on reporter activity. F) Corresponding transcript levels from reporters in panel E.

Due to the palindromic nature of the TCTCGCGAGA motif, we wondered whether the CGCG elements could also activate bidirectional transcription. To test this, we developed a novel bidirectional reporter construct (LuBiDi) to measure promoter activity using firefly and Renilla luciferase genes as reporters of directional transcription from a central control motif (Fig 3A).

We inserted one or two copies of the TCTCGCGAGA motif into the LuBiDi plasmid and measured both reporter activities. A single CGCG element was sufficient to induce both firefly and Renilla reporters whereas two CGCG elements induced an additional 4-fold increase (Fig 3B). To study the motif sequence requirement for this activation, we introduced mutations in the motif that disrupted the wild-type sequence in various locations. First, to determine whether the palindromic structure was more important than the sequence content in conferring the bidirectional transcriptional activity, we exchanged the flanking sequences to form AGACGCGTCT, which maintains both symmetry and CpG content. This mutation abrogated the dual activation of reporters (Fig 3B), suggesting that the promoter function of the CGCG element relies on its sequence polarity. A CGCG → CTCG (underline represents changes) transition mutation (TCTCTCGAGA, reduced CpG content) and an “A” insertion into CGCG (TCTCGACGAGA, unchanged CpG content) abrogated dual reporter activity (Fig 3B). The inclusion of two copies of the A insertion mutant failed to induce transcription. Altogether, these results indicate that the WT element, CGCG core plus the flanking palindromic sequences found in motif 10, are required for promotion of bidirectional transcriptional activity.

To analyze the expression dynamics associated with CGCG elements in single cells, we developed another promoter-less bidirectional reporter (pmCGFP) that codes for enhanced Green Fluorescent Protein (eGFP) and mCherry reporters in opposite directions (Fig A in S2 Fig). One or three copies of TCTCGCGAGA motifs were inserted into this reporter construct, which were then cotransfected into HEK293T cells along with a CMV promoter construct driving the Blue Fluorescent Protein (BFP) as a transfection control. Cells simultaneously expressed both eGFP and mCherry reporter genes starting 12 hours after transfection only for constructs containing the TCTCGCGAGA element (B in S2 Fig). Immunoblot analysis indicated that eGFP and mCherry protein levels were correlated to the number of inserted TCTCGCGAGA motifs (C in S2 Fig). We also tracked individual cells using live imaging microscopy and observed that the two reporter genes are expressed simultaneously after transfection (D in S2 Fig; S1 Video). We also performed a similar imaging experiment using pmCGFP-H2b. This construct is similar to the pmCGFP construct except that the mCherry reporter is fused to the histone H2b protein. Introduction of this reporter construct to the HEK293T and NMuMG mouse mammary cell lines resulted in simultaneous expression of both reporters. Because H2b is a nuclear protein, mCherry signals were also restricted to the nuclei of cells (E and F in S2 Fig). Collectively, these results suggest that this element is a potent bidirectional transcription activator in multiple species.

An analysis of human CGI-associated promoters indicated that CGCG elements could also contain less frequent, single nucleotide variations in TCT or AGA flanking sequences (Fig 3C). To determine the impact of these minor variations on bidirectional transcription activity, we compared LuBiDi constructs with one TCTCGCGAGA motif to those containing naturally variant sequences, using the AGA ↔ TCT flank-exchanged mutant as a negative control (Fig 3D, the variation in a specific nucleotide is underlined). CCT, AGG or ATG flanking sequences decreased relative dual reporter activity whereas variants that contain ATA or TAT showed similar activity to that of the TCTCGCGAGA motif (Fig 3D). The data suggest that some, but not all, variability in the flanking sequences confer promoter activity, albeit at lower efficiencies compared to the TCTCGCGAGA motif. The data also showed that imperfect palindrome elements can still drive bidirectional transcription.

To study the role of copy number variation on bidirectional transcription activity in more detail, we generated LuBiDi reporters that contain one, two or four copies of TATCGCGAGA, a common variant of the CGCG element with an imperfect palindrome. Reporter activity increased proportionally with the number of motifs as measured by luciferase activity or luciferase transcript levels (Fig 3E and 3F).

### Endogenous CGCG elements confer bidirectional transcriptional activity in CGI-associated promoters and methylation abrogates its promoter activity

**Fig 4:**
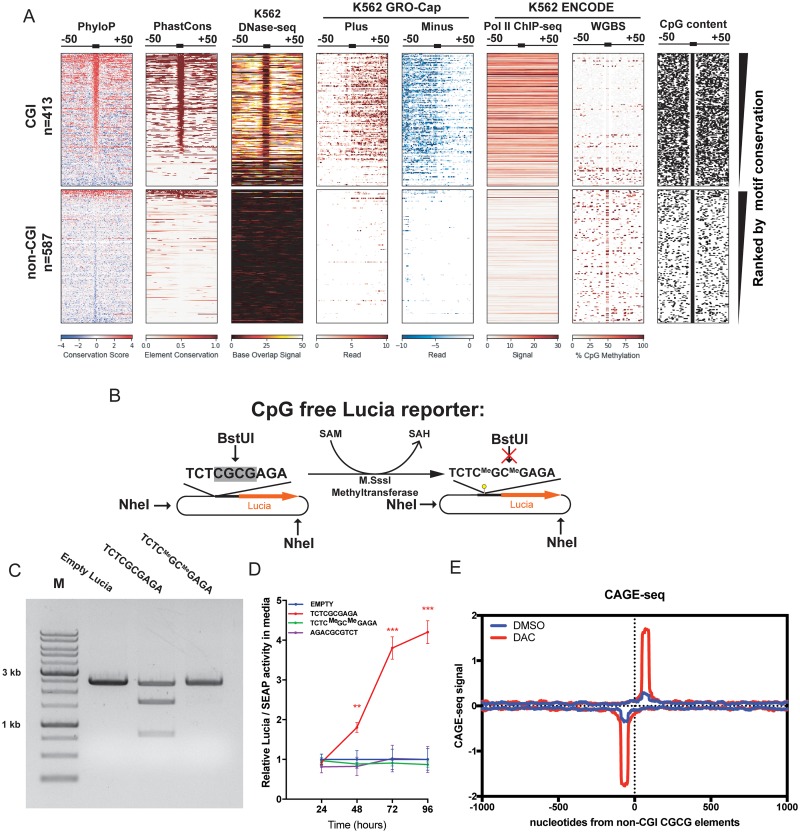
CGCG elements are transcriptionally active in CpG islands and methylation abolishes its activity. A) CGCG elements that occur in CGIs in K562 cells coincide with DNase-seq footprints and associate with divergent plus and minus GRO-Cap transcripts and RNA Pol II occupancy. ENCODE WGBS methylation data show increased methylation of CGCG elements in non-CGIs compared to those in CGIs. B) Methylation of CGCG elements suppresses their promoter activity. Methylation of a TCTCGCGAGA insert in a CpG-free Lucia reporter using M.SssI CpG methyltransferase and SAM. C) Methylation of TCTCGCGAGA in the construct assessed by agarose gel analyses after digestion with NheI plus BstUI (only cuts non-methyl CGCG), which produces a doublet band of 2.3Kb. D) Lucia vs SEAP (transfection control) activity driven by WT, mutant or methylated CGCG inserts in transfected HEK293T cells. Data represent the mean of three replicates +/- SD. E) Strand-specific CAGE-seq signals generated from raw CAGE-seq sequence reads described by Brocks et al. [32] from NCI-H1299 cells treated with either the DNMT inhibitor, DAC, or DMSO control.

To determine if CGCG elements are associated with the *in vivo* bidirectional transcription from endogenous promoters, we analyzed a previously published GRO-cap (global run-on sequencing followed by enrichment for 5′-cap structure) dataset performed on K562 cells [41]. GRO-cap allows for the detection of nascent, often unstable endogenous strand-specific RNA transcripts that are usually undetectable by common RNA-seq methods, likely because of the greatly increased sequencing depth near the transcriptionaly active regions. We found that the bidirectional transcription is associated in the immediate vicinity of the CGCG elements found in CGI-enriched promoters (Fig 4A). Further analysis showed that the endogenous GRO-cap TSS are within 50bp distance of CGCG elements and that CGCG elements are largely positioned in nucleosome depleted regions as determined by the MNase-seq [42] (S3 Fig).

Gene Ontology (GO) analysis showed that genes containing CGCG promoter elements produce protein-coding transcripts whose products form discernible protein-protein interacting networks (S4 Fig). Specifically, these genes encode core components of RNA metabolism and the translational apparatus (Table 1).

**Table 1:** Gene ontology analysis of genes whose promoter contain at least one CGCG element.

Term	p-value	FDR	Enrichment
nuclear-transcribed mRNA catabolic process, nonsense-mediated decay	1.21E-08	1.71E-04	5.45
Co-translational protein targeting to membrane	2.57E-08	1.82E-04	5.96
mRNA metabolic process	1.97E-07	9.26E-04	2.43
amide biosynthetic process	2.77E-07	9.80E-04	3.31
protein targeting to ER	3.26E-07	9.21E-04	5.32
establishment of protein localization to endoplasmic reticulum	4.89E-07	1.15E-03	5.15
protein targeting to membrane	5.69E-07	1.15E-03	4.21
SRP-dependent co-translational protein targeting to membrane	6.11E-07	1.08E-03	5.47
protein localization to endoplasmic reticulum	1.34E-06	2.10E-03	4.75
nuclear-transcribed mRNA catabolic process	3.01E-06	4.25E-03	3.57

Because CpG dinucleotides in CGI-associated promoters are invariably unmethylated [13], we asked if the methylation state of the CGCG elements might explain the observation that only the elements within CGIs are transcriptionally active. Analysis of ENCODE Whole Genome Bisulfate Sequencing (WGBS) from K562 cells indicated that in contrast to CpG-poor regions of the genome, CGCG elements in CGIs are largely unmethylated (Fig 4A). This observation prompted us to determine experimentally whether CpG methylation could alter the promoter activity of the CGCG element. To address this question, we inserted a single copy of TCTCGCGAGA into a secretory luciferase reporter construct that is devoid of CpG sequences (CpG-free Lucia). In this construct, the only CpG sequences are the ones contributed by the CGCG element (Fig 4B). The CpG sequences in this construct were then fully methylated using a CpG methyltransferase that specifically methylates CpG dinucleotides and the results were confirmed by saturated methyl-sensitive enzymatic digestion (Fig 4C). In comparison to the high reporter activity induced by the unmethylated TCTCGCGAGA-containing construct, methylation abrogated the promoter activity (Fig 4D), strongly suggesting that the CGCG methylation antagonizes its promoter function.

To extend these results to endogenous CGCG elements that occur in non-CGI context, we compared CAGE-seq reads of a published dataset from cells treated with either the demethylating agent, deoxyazacitidine (DAC) or vehicle (DMSO) [32]. DAC treated cells showed significant transcriptional activation in regions surrounding CGCG elements located in non-CGI regions on both strands. This observation supports the result of our CpG-free reporter experiment in that CpG methylation of CGCG elements inhibits transcriptional activation, and that demethylation of this element promotes bidirectional transcription *in vivo*(Fig 4E).

A transcription factor zBTB33, also known as Kaiso, was shown previously to be enriched on methylated “CGCG” nucleotides [43]. Kaiso has been shown to interact with the repressive complex SMRT, leading to suppression of gene expression [44]. As illustrated in A of S5 Fig, this transcription factor interacts only with the methylated CGCG element confirming previous observations [45]. The transient overexpression of Kaiso in HEK293T cells did not significantly alter endogenous *DENR* protein levels (B in S5 Fig). These results indicate that Kaiso does not bind to the unmodified (i.e. not methylated) CGCG element. Indeed, ectopic expression of Kaiso does not suppress the *DENR* expression likely because the *DENR* promoter is not methylated *in vivo*. Thus, Kaiso along with other zBTB family members likely only suppress the CGCG element-driven gene expression when this element is methylated.

### The CGCG element activates gene expression in different human promoter configurations

Given that the CGCG element drives bidirectional transcription, we were interested to determine the frequency of this element in annotated uni- vs. bidirectional promoters. The vast majority of CGCG elements (93%) occur in annotated unidirectional promoters that drive coding or long non-coding RNAs, while 7% occur in an annotated bidirectional promoter (Table 2).

**Table 2:** Occurrence of CGCG elements in the annotated human promoters.

Annotated configuration	CGCG elements	Percent
Unidirectional coding	364	80
Bidirectional coding pair	22	5
Unidirectional non-coding	58	13
Non-coding and coding pair	9	2

**Fig 5:**
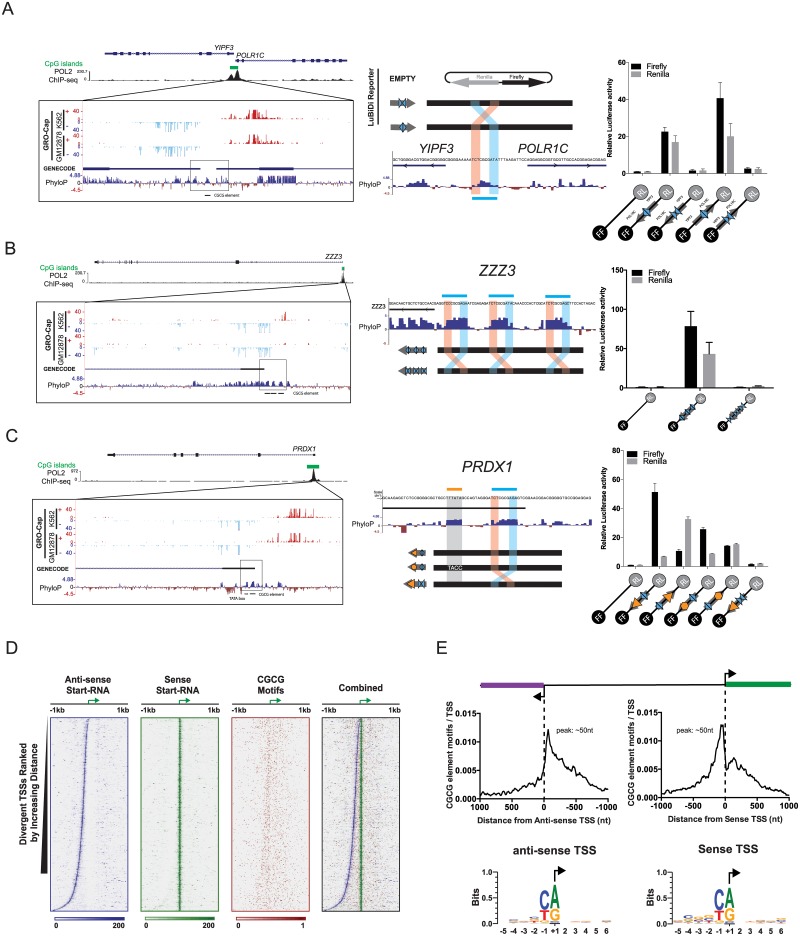
CGCG elements in CGI promoters drive gene expression. A) The bidirectional promoter driving the *POLR1C*/*YIPF3* genes (left panel) contains a conserved CGCG element between annotated TSSs (middle), which was cloned and tested for bidirectional promoter activity in LuBiDi. Right panel- the promoter insert orientation is defined by a large grey arrow, with the WT CGCG element flank orientation represented by ◊, and the flank-exchanged variants represented by ⊳⊲. B) The *ZZZ3* promoter contains three CGCG elements. Although this promoter is annotated as unidirectional, the GRO-Cap analysis indicated associated divergent transcripts on the opposite strand. C) The promoter of *PRDX1* gene contains both a TATA-box (⊳) and a CGCG element. D) Start-seq data analysis of CGCG elements in the mouse genome. The results suggest that CGCG elements are positioned in the nucleosome depleted regions between sense and anti-sense TSS. E) CGCG elements occur mostly within 50bp of sense and anti-sense Start-seq TSSs. Sense and anti-sense TSS occur in Py/Pu dinucleotide motif where Pu is the putative +1 nucleotide. Data are represented as the mean of three replicates +/- SD.

However, recent studies suggest that the majority of what were classically defined as unidirectional promoters produce unstable “promoter upstream transcripts” (PROMPTS) [46]. Based on this, we investigated the role of CGCG elements in three different endogenous promoters that differ in their annotated directionality and whether they combine CGCG element with TATA-boxes. In order to determine the role of endogenous CGCG elements, we simultaneously disrupted CGCG elements but maintained CG content by exchanging the flanking sequences (i.e. TCTCGCGAGA → AGACGCGTCT). We first focused on the *POLR1C*/*YIPF3* bidirectional promoter region, which has divergent TSS separated by 30 nucleotides that flank a single CGCG element. We inserted a promoter fragment (30bp) containing the wild-type CGCG element into the LuBiDi construct, and as a comparison, constructs were generated in which the flanking sequences (AGA and TCT) were exchanged. The WT fragment from *POLR1C*/*YIPF3* promoter induced bidirectional expression irrespective of its orientation (Fig 5A). In contrast, the flank-exchanged mutants, regardless of insert orientation, did not show any discernible reporter activity.

Next, we analyzed the *ZZZ3* promoter which is similar to the *DENR* promoter in that it contains three CGCG elements (Fig 5B). Although the promoter is annotated as directional, PROMPTs on the opposite strand in both the K562 and GM12878 GRO-Cap datasets were found (Fig 5B, UCSC genome browser plot). To determine whether these elements are responsible for the divergent *ZZZ3* transcripts, we inserted CGCG elements or flank-exchanged elements from the *ZZZ3* promoter into a LuBiDi construct. As shown in Fig 5B, WT sequences but not flank-exchanged could induce bidirectional reporter expression. An analysis of the *DENR* promoter also showed that their three CGCG elements drive bidirectional transcription in the LuBiDi assay, and that disruption of CGCG core sequences with A insertions abrogated the bidirectional promoter activity (S6 Fig).

We also studied the *PRDX1* promoter, a rare example in which both a single CGCG element plus a TATA-box map within the CpG-enriched promoter [47]. An analysis of GRO-Cap datasets indicated a predominant TSS approximately 25 nucleotides downstream of the TATA-box (Fig 5C), yet divergent transcripts were found starting roughly 50-70 bp upstream of the coding region in both K562 and GM12878 cells. To investigate the role of the TATA-box in this configuration, we inserted a fragment containing the TATA-box and CGCG element from this promoter into LuBiDi. We also produced mutants including one that disrupted the first TA in the TATA-box with CC sequences and another in which the TATA-box orientation was reversed relative to the CGCG element. The WT *PRDX1* promoter fragment mainly drove unidirectional downstream transcription (Fig 5C) although some opposite direction reporter activity was noted. Mutation of the TATA-box severely attenuated downstream directional promoter activity (Fig 5C). Interestingly, the reporter containing a flank-exchanged CGCG element did not show any reporter activity even in the presence of a WT TATA-box, suggesting that the CGCG element not only promotes divergent transcription but also acts as a required activator for the TATA-box in this promoter.

To further study the role of CGCG elements in the context of bidirectional promoters, we analyzed a set of mouse bidirectional promoters previously defined using Start-seq [48]. We assessed the presence of CGCG elements throughout the intervening regions in such bidirectional promoters. The coupled sense/anti-sense TSS form boundaries that flank a nucleosome-depleted region, characterized by an open chromatin structure that permits high accessibility for transcriptional machinery (Fig 5D). This analysis indicated that although CGCG elements do not show a fixed distance to sense or anti-sense Start-seq TSSs, they mostly positioned within 50 nucleotides of the predominant TSS in mouse bidirectional promoters. Associated TSS occur in context of PyPu motifs where Py and Pu are -1 and +1 nucleotides, respectively. (Fig 5E).

### CGCG elements promote transcription through divergent TSS

**Fig 6:**
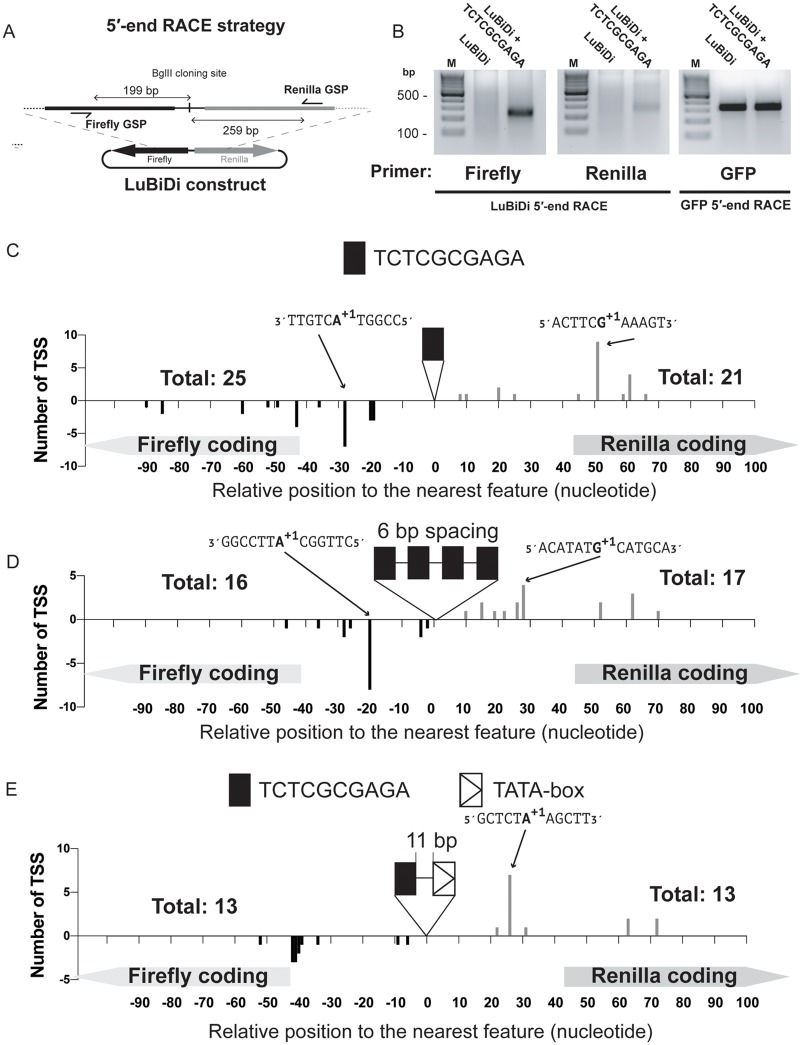
CGCG element is associated with divergent transcription start sites. A) The location of gene-specific primers used in 5′-RACE experiments to identify bidirectional TSSs in the LuBiDi based reporter constructs. Firefly and Renilla luciferase primers were designed 199 and 259 bp away from the BglII cloning site, respectively. B) Agarose gel image of firefly and Renilla luciferase RACE PCR products for the LuBiDi constructs containing none or one copy of TCTCGCGAGA. GFP transcripts were used as an internal control for RACE. C) TSS were determined for the LuBiDi construct containing a single copy of the TCTCGCGAGA motif. The sequence of +1 nucleotide and flanking five nucleotides are also shown on major TSSs. D) Bidirectional TSS associated with the LuBiDi construct containing four tandem copies of TCTCGCGAGA. E) Divergent TSS for the LuBiDi construct that contained a TATA-box and a CGCG element from the *PRDX1* promoter. TSS positions are indicated in nucleotide relative to the nearest feature (the CGCG element or the TATA-box). The number of sequenced clones for each reporter constructs are indicated above coding regions.

Previously identified promoter elements such as the TATA box and the TCT motif promote transcription through a focused putative TSS that occurs either at a fixed distance downstream (in the case of TATA box) or on a specific nucleotide within the element in the case of the TCT motif [49]. To map the bidirectional TSSs associated with the CGCG element in both directions, we employed 5′-end RACE (Rapid Amplification of cDNA Ends) strategy using RNA extracted from HEK293T cells transfected with LuBiDi reporter constructs along with pEGFP as a transfection control (Fig 6A). This robust method has been successfully used to determine the TSS of many genes in human and other organisms previously [50, 51].

As shown in Fig 6B, 5′-end RACE analysis of RNA from cells transfected with the promoterless LuBiDi construct (A in S7 Fig) did not produce transcripts in either direction, suggesting that the reporter construct does not exhibit intrinsic transcriptional activity. In contrast, 5′-end RACE produced major products for firefly and Renilla transcripts from a LuBiDi construct containing one copy of the TCTCGCGAGA motif (B in S7 Fig). Sequencing of the RACE products from both firefly and Renilla transcripts showed a strong preference for purines as the +1 nucleotide, and pyrimidines as the -1 nucleotide, conforming to previous genome-wide observations [47]. Although multiple TSSs were found in the sense or anti-sense directions, there was a predominant firefly luciferase TSS (7 of 25 clones) 28 nucleotides and a predominant Renilla luciferase TSS (9 of 21 clones) 51 nucleotides from the TCTCGCGAGA element (Fig 6C). However, we observed that a majority of preferred Renilla TSS were downstream of the initiation codon (ATG), and thus, unlikely to produce active Renilla luciferase product. This likely explains why the relative Renilla luciferase activity, but not the transcript level, is always lower than that of the firefly luciferase, as was previously observed in Fig 3E and 3F.

We also used 5′-RACE to determine TSS in the reporter construct containing four tandem copies of TCTCGCGAGA motif (B in S7 Fig). As shown in Fig 6D, no TSS was observed in TCTCGCGAGA or in the 6 nucleotide spacer (BglII site) regions between the four elements. In contrast to the results observed with the construct containing one CGCG element, TSS were relocated closer to CGCG elements likely as a result of a potential cooperative Pol II recruiting mechanism when CGCG elements occur in tandem copies.

Next, we determined how the presence of a TATA-box affects CGCG element-driven TSS choice using the LuBiDi reporter containing these elements from the *PRDX1* promoter (B in S7 Fig). In this construct, the TATA-box was arranged between the Renilla reporter and the CGCG element. Sequencing of the Renilla RACE products showed a predominant TSS (7 of 13 clones) 26 nucleotides downstream of the TATA box on the Renilla-coding strand (Fig 6E). In contrast, on the firefly reporter coding strand, there was a concentration of multiple TSSs 40-43 nucleotides downstream of CGCG element. This TSS pattern differs from those induced from the construct containing just a single copy of the CGCG element (Fig 6C). Together with reporter data presented in Fig 5C, these results suggest that the CGCG element and TATA box cooperate to induce transcription in the *PRDX1* promoter.

## Discussion

In this study, we identify a novel promoter element that drives bidirectional transcription mainly in the context of TATA-less promoters. Although in previous studies, sequences similar to this element were found in the promoter of individual genes, the functional role of the CGCG element in CGIs and TATA-less promoters in the human genome was never explored [22–25]. Whereas other promoter elements (e.g. TATA and GC boxes) require an activator binding site to initiate directional transcription [6], a single instance of the CGCG element is both necessary and sufficient to promote bidirectional transcription. However, in comparison to other known promoter elements that induce transcription, which typically occur once in most promoters, CGCG elements occur in multiple copies in a small percentage of CGI-containing promoters, a phenomenon that likely influences RNA polymerase recruitment and subsequent transcriptional rates.

An interesting yet poorly studied feature of vertebrate genomes is the presence of CpG-rich regions known as CGIs [14]. Although CGIs mark transcriptionally active regions of the genome, the mechanism of RNA polymerase recruitment in these regions has been elusive [13]. Through enrichment analysis, we found that CGCG elements are enriched in a group of CGI-containing promoters and that they can recruit transcriptional machinery to promote bidirectional transcription, a feature common among transcriptionally active CpG islands [19]. Additionally, we provide evidence that in some rare cases, the CGCG element could interact functionally with an adjacent TATA-box to activate directional gene expression. Similar synergistic activities have been described previously [52, 53] suggesting that the CGCG element also shares this attribute with other known promoter elements.

In previous studies, a motif called hDRE (TGTCGYGAYA, where Y is a pyridimide nucleotide) was shown to positively activate metabolism-related and ribosomal protein genes through binding of ZBED1(also known as DREF) protein [54, 55]. The sequence of hDRE is very similar to that of CGCG element. However, as we showed in Fig 3C, TCTCGCGAGA and TATCGCGAGA (underlined nucleotides denote deviations from the hDRE sequence) sequences that account for the majority of CGCG elements do not overlap with the hDRE (a list of human genes that are potentially regulated by the CGCG element is provided in the S2 File). As outlined in the S5C and S5D Fig, successful knockdown of ZBED1 using specific lentivirus-induced shRNAs do not change the endogenous protein or transcript levels of the *DENR* gene whose promoter contains three copies of the CGCG element. This result suggests that ZBED1 may not regulate the CGCG element-driven transcription of genes from the TATA-less CpG islands-associated promoters. Therefore, we speculate that the CGCG element is a different promoter element from the hDRE motif that function through transcription factor ZBED1 and is regulated by different mediators.

How housekeeping genes whose products are core components of cellular processes are transcriptionally regulated is poorly understood. In this study, we found that genes whose products play a central role in translation and transcription are enriched for CGCG elements in their CGI-associated promoters. This analysis led us to identify a group of ribosomal genes whose CpG rich promoters contain one or multiple CGCG elements (A in S8 Fig). As shown in the panel B of S8 Fig, a group of ribosomal protein promoters containing CGCG elements do not coincide with the previously described TCT motif that is thought to regulate the transcription of this gene class in humans [49]. These results suggest that TCT and CGCG elements either cooperate or act independently to regulate the expression of specific sets of ribosomal protein genes in the human genome. In addition to genes encoding ribosomal proteins, promoters of key translation initiation factor genes encoding *EIF5*, *EIF3H*, and *DENR*, as well as the essential translation termination factor *ETF1*, contain CGCG elements in their promoters. This is consistent with the current perspective that different classes of promoter elements regulate functionally distinct protein coding genes [1].

Additionally, we demonstrated that methylation of CpGs in the CGCG element could suppress promoter activity. Indeed, roughly 80 percent of CpG sites in the genome, particularly CpGs that occur outside of CGIs, are methylated [56]. We speculate a switch-like mechanism that could activate or repress gene expression based on the methylation status of CGCG elements. Accordingly, we propose a model where CGCG elements, when occurring in CGIs, are protected from methylation thereby maintaining promoter activity in housekeeping genes. In contrast, CGCG elements in other regions of the genome would be more subject to methylation, resulting in transcriptional silencing. In theory, DNA methylation of CGCG elements could protect the genome from spurious transcription, as reviewed elsewhere [57]. A similar switch-like mechanism for a group of transcription factors that contain CpG motif has been described in the past in which CpG methylation would affect the binding affinity of transcription factors [58]. Although the nature of the factor, or factors, that bind to non-methyl CGCG element has yet to be clarified, our results suggest that ChIP-seq studies should be interpreted with greater consideration to account for the differential binding of proteins to methyl or non-methyl CpG-containing motif sequences.

## Conclusion

In this study, we provide strong evidence that CGCG elements are evolutionarily conserved in vertebrates, functioning as an active component of CGI-associated promoters. The unmethylated form of the element may be sufficient to drive bidirectional transcription of TATA-less promoters. An interesting, yet very important question to address in future studies is whether the CGCG element functions as a core promoter element or as a sequence-specific transcription factor binding site (SSTFBS). An argument for the core promoter element characteristic of the CGCG element includes its ability to initiate local *de novo* bidirectional transcription in the absence of a core promoter element from nearby Py/Pu(+1) sites. This notion is supported by studies that show that SSTFBSs cannot initiate transcription in the absence of a core promoter element. However, similar to SSTFBSs, CGCG elements can occur in tandem copies and the copy number modulates transcription intensity. Unlike traditional core promoter elements, the CGCG element is not positioned at a fixed distance to TSSs, but it is found within a range of 20 to 70 nucleotides (with a peak of apporoximatly 50 nucleotides) upstream of TSS on either strands as was determined by the analysis of Start-seq and GRO-Cap datasets. It is also conceivable that CGCG elements modulate the induction of transcription by recruiting factors that could affect nucleosomal positioning in CGIs. Identification of the CGCG element interacting factor will likely clarify whether the CGCG element functions as a core promoter element and its role in driving transcription of housekeeping genes from CpG-rich and TATA-less promoters.

## Supporting information

S1 Fig The CGCG element (motif 10) is associated with DNase-seq footprint in different cell lines. A) ENCODE DNase-seq footprints of motif 7 and 10 for available cell lines. B) TCTCGCGAGA motif occurs within 50bp of annotated TSSs in the human and mouse genomes.
(EPS)

S2 Fig The CGCG elements promote simultaneous expression of GFP and mCherry genes in the pmCGFP reporter construct. A) The pmCGFP bidirectional reporter structure. B) Fluorescence image 24h after transfection of HEK293T cells with pmCGFP constructs containing 0, 1 or 3 copies of the TCTCGCGAGA motif. CMV-driven BFP expression was used as an internal control. C) Immunoblots showing levels of GFP and mCherry expression 24 and 48h post transfection. D) Time-lapse imaging of HEK293T cells transfected with pmCGFP containing three copies of TCTCGCGAGA for 24h shows that both reporters are simultaneously expressed. Scale bar is 100 *μ*m. E) CGCG element confers bidirectional expression of GFP and H2b-mCherry reporter genes in HEK293T. Time-lapse images of HEK293T cells transfected with pmCGFP-H2b (h2b-mCherry fused gene). Please note delayed H2b-mCherry signals as the fused mCherry protein is being trafficked into the nucleus. F) Images of NMuMG mouse cells transfected with a pmCGFP-H2b construct containing either 3 copies of the wild-type TCTCGCGAGA motif or 3 copies of the TCTCTCGAGA mutant motif.
(EPS)

S3 Fig GRO-cap profiles were generated for +/- 1000bp from TCTCGCGAGA and TATA-box sites in K562 cells. The GRO-cap signals, which map TSS *in vivo*, clearly show TSS peaks are located 50bp from the TCTCGCGAGA in both directions. The figure also includes MNase-seq plots for the two promoter element +/-1000bp. The data indicates that genomic regions containing TCTCGCGAGA and TATA-boxes are nucleosome depleted.
(EPS)

S4 Fig REACTOME gene network analysis of CGCG containing promoters. An analysis of genes that contain CGCG elements in their promoters found that most of these genes can be clustered into distinct functional groups as indicated in the figure.
(EPS)

S5 Fig Kaiso binds specifically to the methylated CGCG element. A) Pulldown experiment using biotin-tagged CGCG oligonucleotides followed by immunoblotting for Kaiso or tubulin. B) Immunoblot analysis of transient expression of Kaiso in HEK293T cells. Two independent replicates (Rep1 and Rep2) were used in this experiment. C) Successful knockdown of ZBED1 does not change the DENR protein product. D) RT-PCR analysis of ZBED1 knockdown cells does not attenuate the transcript levels of the *DENR* gene.
(EPS)

S6 Fig CGCG elements in the DENR promoter promote divergent transcription. A) GRO-cap signal tracks of the CpG-rich DENR promoter. B) The CGCG elements in the DENR promoter, regardless of the insert direction, activated bidirectional reporter luciferase activities. Insertion of an “A” in the center of CGCG elements eliminated the promoter activity. Data are represented as the mean of three replicates ± SD.
(EPS)

S7 Fig Sequence of LuBiDi constructs used for RACE experiments. A) The sequence flanking the BglII restriction site where DNA duplexes were inserted into the promoterless LuBiDi construct. The reporter genes in either direction are shown in bold. B) The sequences of DNA fragments inserted into LuBiDi, containing flanking BglII restriction sites (boxed), used for RACE analyses.
(EPS)

S8 Fig CGCG elements are enriched in ribosomal protein promoters. A) Aligned sequences of CGCG elements and flanking regions in the promoters of ribosomal proteins genes, with the resulting consensus sequence of TCTCGCGAGA shown below. The list also indicates whether the TCT or hDRE motifs are present in a given promoter. B) Venn diagram showing the distribution of TCT and CGCG elements in human ribosomal proteins promoters.
(TIF)

S1 Video CGCG elements activate dual expression of GFP and mCherry reporter genes. Live cells were transfected with pmCGFP reporter constructs containing none, one or three tandem copies of TCTCGCGAGA. Cells were incubated in a chamber supporting normal cellular growth and images were captured to record mCherry and GFP signals every 30 minutes. The top panel for each reporter assay shows overlapping signals. Lower panels show GFP or mCherry fluorescence channels.
(MP4)

S1 File Contains sequence of oligonucleotides used in this study. (PDF)

S2 File Contains a list of human genes that are potentially regulated by CGCG elements. (TXT)
