# Supplementary figures and images for: A methyl-sensitive element induces bidirectional transcription in TATA-less CpG island-associated promoters
Source: PLoS One. 2018 Oct 17;13(10):e0205608. doi: 10.1371/journal.pone.0205608 (PMC6192621; doi:10.1371/journal.pone.0205608)

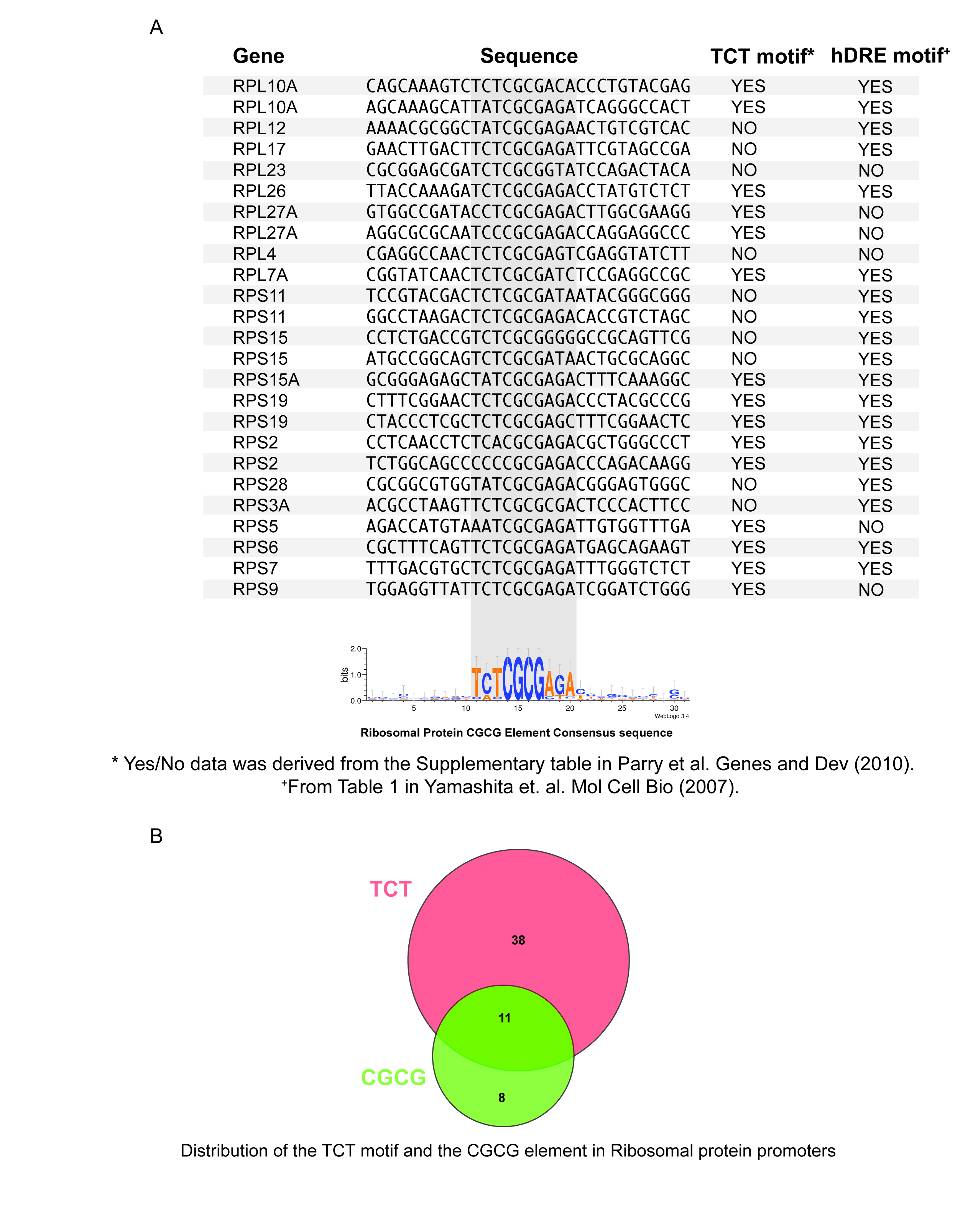

Supplement: S8 Fig — A) Aligned sequences of CGCG elements and flanking regions in the promoters of ribosomal proteins genes, with the resulting consensus sequence of TCTCGCGAGA shown below. The list also indicates whether the TCT or hDRE motifs are present in a given promoter. B) Venn diagram showing the distribution of TCT and CGCG elements in human ribosomal proteins promoters. (TIF) [file pone.0205608.s008.tif]
