# Supplementary material for: A methyl-sensitive element induces bidirectional transcription in TATA-less CpG island-associated promoters
Source: PLoS One. 2018 Oct 17;13(10):e0205608. doi: 10.1371/journal.pone.0205608 (PMC6192621; doi:10.1371/journal.pone.0205608)
Supplement: S1 File — (PDF) [file pone.0205608.s010.pdf]

Table 1. Sequence of oligonucleotides cloned into pGL2-Basic reporter from the DENR Promoter (cloned into pGL2-basic BglII and MluI)

Name	Sequence
CGCG #1	CGCGTCTTCTCGCGACACGCTGA
CGCG #1, 2	CGCGTCTTCCGGCTTCTCGCGACATCCGCCCAGCGGCGGCCGGAATCTCGCGATACGCTGA
CGCG #1, 2, 3	CGCGTCTTCCGGCTTCTCGCGACATCCGCCCAGCGGCGGCCGGAATCTCGCGATAAAGGCTCATTGTCTCGCGGGAGCGCTGA
CTCG #1, 2, 3	CGCGTCTTCCGGCTTCTCtCGACATCCGCCCAGCGGCGGCCGGAATCTCtCGATAAAGGCTCATTGTCTCtCGGGAGCGCTGA
Motif 10 (BglII)	GATCTTCTCGCGAGA
Motif 7 (BglII)	GATCTACTACAATTCCCA

Table 2. Primer sequence to clone firefly from pGl2-basic into pRL2-null (cloned into BglII site[GATCT]

Name	Sequence
Forward	GAGATCTAAGTAAGCTTGGCATTCCG
Reverse	AAGATCTGACGATAGTCATGCCCCGCG

Table 3. Site-directed mutagenesis primers used to remove the secondary BglII introduced during the cloning:

Name	Sequence
Forward	CACATGGCTCGACATATCTGACGATAGTCA
Reverse	TGACTATCGTCAGATATGTCGAGCCATGTG

Table 4. DENR Promoter fragments:

Name	Sequence
CGCG #1	GATCTCGGCTTCTCGCGACAA
CGCG #1, 2	GATCTCGGCTTCTCGCGACATCCGCCCAGCGGCGGCCGGAATCTCGCGATAA
CGCG #1, 2, 3	GATCTCGGCTTCTCGCGACATCCGCCCAGCGGCGGCCGGAATCTCGCGATAAAGGCTCATTGTCTCGCGGGAGCA
CGACG #1, 2, 3	GATCTCGGCTTCTCGACGACATCCGCCCAGCGGCGGCCGGAATCTCGACGATAAAGGCTCATTGTCTCGACGGGAGCA

Table 5. PRDX1 Promoter fragments

Name	Sequence
WT CGCG + WT TATA	GATCTGGGGCGCTGCCTTTATAGCCAGTAGGGATCTCGCGAGAGTCGGAA
REV CGCG + WT TATA	GATCTGGGGCGCTGCCTTTATAGCCAGTAGGGAAGACGCGTCTGTCGGAA
WT CGCG + MU TATA (CCTA)	GATCTGGGGCGCTGCCTTCCTAGCCAGTAGGGATCTCGCGAGAGTCGGAA

Table 6. ZZZ3 Promoter fragments

Name	Sequence
WT	GATCTTCCCGCGAGAATCGAGAGATCTCGCGATACAAACCCACTCGCATCTCGCGAGCTA
Flank-exchanged mutant	GATCTAGACGCGTCCATCGAGAGAATACGCGTCTCAAACCCACTCGCAAGCCGCGTCTTA

Table 7. POLR1C/YIFP3 Promoter fragments

Name	Sequence
WT CGCG	GATCTACGGGGGCGGGGAAAAATCTCGCGATATTTAAGATTCCAGGAGGCGGTGCGTTGCCACGGAGACGGAA
Flank-exchanged CGCG	GATCTACGGGGGCGGGGAAAAAATACGCGTCTTTTAAGATTCCAGGAGGCGGTGCGTTGCCACGGAGACGGAA

Table 8. CGCG variations

Name	Sequence
WT	GATCTTCTCGCGAGA
A-insert mutant	GATCTTCTCGACGAGAA
CCTCGCGAGA	GATCTCCTCGCGAGAA
TCTCGCGATG	GATCTTCTCGCGATGA
TCTCGCGATA	GATCTTCTCGCGATAA

Table 9. pmCGFP-H2b Reporter (pcil cloning site)

Name	Sequence
Forward	gacatgtCCACCATGCCTGAACCCTCTAAGTCTGC
Reverse	tcacatgtCCATAGAGCCCACCG
WT	CCGGTTCTCGCGAGA
Flank-exchanged mutant	CCGGTTCTCGCGAGA

Table 10. CpG-free Lucia (HindIII cloning site)

Name	Sequence
WT	AGCTTTCTCGCGAGAA
Flank-exchanged	AGCTTAGACGCGTCTA

Table 11. qPCR primers

Name	Forward	Reverse
Firefly	ATAAATAACGCGCCCAACAC	AGAGATACGCCCTGGTTCCT
Renilla	GTGGTGGGCCAGATGTAAAC	ATTTGCCTGATTTGCCCATA
AmpR	TTTGCCTTCCTGTTTTTGCT	ATAATACCGCGCCACATAGC
DENR	ACAGTGCCAAGTTAGATGCCG	TCCTTGACCCTCACTAATTCCA
HPRT1	ACCAGTCAACAGGGGACATAA	CTTCGTGGGGTCCTTTTCACC

Table 12. ChIP primers

Name	Forward	Reverse
LuBiDi Reporter	TACCAACAGTACCGGAATGC	CGACCAACTTCTGCAGTACC
GAPDH	CGGCTACTAGCGGTTTTACG	AAGAAGATGCGGCTGACTGT

Table 13. CRISPR/Cas9 guide sequences

Name	Sequence
sgRNA1	GTCGCGAGAAGCCGGAAGAC
sgRNA2	ATAAAGGCTCATTGTCTCGC

Table 14. PCR primers to screen CRISPR-edited *DENR* promoter

Name	Forward	Reverse
DENR	TGCGTCTGCAAGTCACGTTC	CACTCTCTACGAGCTTCTCGG

Table 15. RACE Primers

Name	Sequence
Firefly	GATTACGCCAAGCTTCCGCGTACGTGATGTTCACCTCGAT
Renilla	GATTACGCCAAGCTTACCGCGCTACTGGCTCAATATGTGG
GFP	AAGTCGTGCTGCTTCATGTG
Universal Primer Mix long	ctaatacgactcactatagggcAAGCAGTGGTATCAACGCAGAGT
UPM short	ctaatacgactcactatagggc
